# Identification of a biomarker panel for colorectal cancer diagnosis

**DOI:** 10.1186/1471-2407-12-43

**Published:** 2012-01-26

**Authors:** Amaia García-Bilbao, Rubén Armañanzas, Ziortza Ispizua, Begoña Calvo, Ana Alonso-Varona, Iñaki Inza, Pedro Larrañaga, Guillermo López-Vivanco, Blanca Suárez-Merino, Mónica Betanzos

**Affiliations:** 1 GAIKER Technology Centre, Parque Tecnológico, Edificio 202, 48170 Zamudio, (Bizkaia), Spain; 2 Computational Intelligence Group, Departamento de Inteligencia Artificial, Universidad Politécnica de Madrid, Campus de Montegancedo, 28660 Boadilla del Monte, Spain; 3 Medical Oncology Service, Hospital de Cruces, Plaza de Cruces s/n, 48903 Barakaldo, (Bizkaia), Spain; 4 Department of Cell Biology and Histology, School of Medicine and Dentistry, University of the Basque Country, 48940 Leioa, (Bizkaia), Spain; 5 Department of Computer Science and Artificial Intelligence, Computer Science Faculty, University of the Basque Country, 20018 San Sebastián, (Gipuzkoa), Spain

## Abstract

**Background:**

Malignancies arising in the large bowel cause the second largest number of deaths from cancer in the Western World. Despite progresses made during the last decades, colorectal cancer remains one of the most frequent and deadly neoplasias in the western countries.

**Methods:**

A genomic study of human colorectal cancer has been carried out on a total of 31 tumoral samples, corresponding to different stages of the disease, and 33 non-tumoral samples. The study was carried out by hybridisation of the tumour samples against a reference pool of non-tumoral samples using Agilent Human 1A 60-mer oligo microarrays. The results obtained were validated by qRT-PCR. In the subsequent bioinformatics analysis, gene networks by means of Bayesian classifiers, variable selection and bootstrap resampling were built. The consensus among all the induced models produced a hierarchy of dependences and, thus, of variables.

**Results:**

After an exhaustive process of pre-processing to ensure data quality--lost values imputation, probes quality, data smoothing and intraclass variability filtering--the final dataset comprised a total of 8, 104 probes. Next, a supervised classification approach and data analysis was carried out to obtain the most relevant genes. Two of them are directly involved in cancer progression and in particular in colorectal cancer. Finally, a supervised classifier was induced to classify new unseen samples.

**Conclusions:**

We have developed a tentative model for the diagnosis of colorectal cancer based on a biomarker panel. Our results indicate that the gene profile described herein can discriminate between non-cancerous and cancerous samples with 94.45% accuracy using different supervised classifiers (AUC values in the range of 0.997 and 0.955).

## Background

Colorectal cancer (CRC), is the third most common form of cancer and the second leading cause of death among cancers worldwide, with approximately 1, 000, 000 new cases of CRC and 50, 000 deaths related to CRC each year [1,2]. Sporadic colon cancer represents the 70% of newly diagnosed cases, and it is believed to slowly develop via a progressive accumulation of multiple mutations that affect tumour suppressor genes, as well as oncogenes or mismatch repair genes (MMR) [3].

Statistics concerning colon cancer survival show differences between countries. In US, the overall five-year survival rate is 62% while in Europe is 43%. The reasons for this different behaviour are not very clear, although quality of care and screening programs could play a central role in the survival of CRC, since it is well established that the stage of the disease at diagnosis greatly impacts colon cancer survival rates. In this way, the US Centres for Disease and Control Prevention (CDC) state that the 5-year survival rate for persons who received a diagnosis of localized colorectal cancer is 91% compared with 70% for regional-state cancer and 11% for distant -stage cancer [4]. Also, a study registered at the National Cancer Institute's SEER database, conducted with more than 28, 000 people diagnosed with colon cancer between 1998 and 2000, found that the observed 5-year survival rates related to the stage of the disease at diagnosis were the following: I-74%, IIA-67%, IIB-59%, IIC-37%, IIIA-73%, IIIB-46% and IIIC-28% (source: American Cancer Society).

This and other evidences have convinced the scientific and medical community of the great importance of screening for CRC to reduce incidence and mortality, through detection of premalignant polyps as well as diagnosis of early -stage cancer [4,5]. As a result, data from the CDC show that CRC incidence and mortality have experienced a decline in recent years due to the screening campaigns [6,7]. In spite of this, the same studies indicate that CRC remains the second most common cause of cancer deaths after lung cancer in the US and the leading cause of cancer deaths among non-smokers. In this context, there is a global awareness for the implementation of CRC screening programmes [8]. Not only the US, but also France put into action a screening programme in 2003, Finland in 2004, UK in 2006, etc. However, there is no international consensus on the preferred strategy to carry on the screening, mainly due to the limitations of the available screening techniques at present.

The currently used methods for the early detection of CRC are the Faecal Occult Blood Test (FOBT) and the endoscopy. FOBT is simple, inexpensive and the least invasive method of screening available. Also, it has been shown through prospective randomized trials that FOBT reduces CRC mortality, and consequently the evidence for its use is robust. However, FOBT presents relatively high false negative and false positive rates, and it has particularly poor sensitivity for the detection of early-stage lesions [9-11]. In an attempt to improve on the false positive rates of FOBT, a new Faecal Immunochemical testing (FIT) has been developed. It has slightly superior performance characteristics but at a greatly increased financial cost, and its implementation has not been effective as yet [12].

On the other hand, colonoscopy offers significant improvements in detection rates for CRC but it also has important disadvantages associated, as inconvenience, high economic burden and potential major complications (bleeding, perforation) [13,14].

All this emphasizes the urgent necessity of new diagnostic approaches in order to improve the outcome of CRC screening programs. In particular. there is a clinical need for identifying specific biomarkers for early detection of CRC [2,12]. Moreover, there is at the present time a widespread awareness, not only between scientists and practicing clinicians but also among regulatory organizations and healthcare systems, that the development of biomarkers will offer the major advances in CRC detection [15]. The scientific world even holds an expectation that a new generation of molecular markers should improve compliance with CRC screening in the same way other markers do in other illnesses screening programs (lipid monitoring, PSA,...).

The recent advances in genomics and proteomics have contributed to our molecular understanding of CRC by evaluating the expression profiles of genes and proteins, in cancerous and non-cancerous surrounding tissues and body fluids. The identification of genes and/or proteins that are characteristic of the development of CRC can render potential biomarkers that will facilitate the early detection of CRC. There is quite a number of recently discovered potential molecular biomarkers, such as CEA, CA 19-9, K-*ras*, L-DNA, APC, TIMP-1, NNMT, MIF, PSME3, Septin 9, MMP-9, MMP-7, Spondin-2, DcR3, Trail-R2, MICI, CCSA-2, CCSA-3, CCSA-4, etcetera [16]. Some of them have been questioned because of insufficient sensitivity or specificity (CEA, CA 19-9,...), others because of poor performance in early stages of CRC (TIMP-1,...), and some others remain promising but there is insufficient evidence for their routine implementation (CCSA-3, CCSA-4, MIF, DcR3, Spondin-2,...) [17,18]. Many authors agree that a panel of biomarkers will be likely necessary to reach appropriate sensitivity for clinical use as a screening biomarker, due to the genetic heterogeneity of CRC [12,19]. Furthermore, a limited number of markers have been identified to date in CRC, but their individual use has led to conflicting results [1]. In this context, genomic techniques, such as DNA microarrays, allow high-throughput analysis of genes, rendering big volumes of data which increases the possibilities for uncovering potential biomarkers. Namely, DNA microarray-based gene expression profiling technology provides a strategy to search systematically with a combinatorial manner for molecular markers of colon cancer.

Our aim in the present study was to develop a model or biomarker for the objective diagnosis of CRC based on gene expression patterns. For this purpose, we used the microarray technology in combination with advanced statistics analysis techniques. The identification of a robust panel of CRC-specific biomarkers through genomics would be the cornerstone for their posterior development into non invasive samples-based diagnosis markers.

## Methods

### Patients samples

A total of 64 tissue samples--33 non-tumoral tissues (NT) and 31 tumoral tissues (T)--were obtained from patients with CRC diagnosed at different stages in Cruces Hospital (BIOEF) after informed consent. Ethical approval was obtained from the Clinical Research Ethics Committee of University Cruces Hospital (reference number CEIC E02/27).

After surgery, an anatomopathologic analysis was carried out to confirm diagnosis as well as tumour staging, using TNM and Dukes classifications. In TNM system, stage is expressed in roman numerals from stage I (the least advanced) to stage IV (the most advanced) and some stages are subdivided with letters. Dukes classification (adapted by Astler and Coller) is an older staging system that groups patients into either Stage A, B, or C depending on the extent of the cancer- localized, spread through the intestinal wall and metastasis to lymph nodes. Stage D was later added to indicate evidence of metastases.

None of the patients received neoadjuvant therapy prior to operation. All the samples were collected in a tube containing RNA*later *solution (Qiagen) to preserve the RNA from degradation and kept at -80°C until future use. Clinical features of selected patients are listed on Additional file 1.

### Experimental design

To search for genetic markers, the experimental design comprised the hybridisation of each sample (both tumoral and non-tumoral samples) against a reference pool consisting of the non-tumoral samples [20]. In this sense, a total of 31 microarrays were hybridised comparing tumoral samples and the pool. In addition, to reduce the background noise due to variability among non-tumoral samples, each non-tumoral sample was hybridised against the reference pool. These comprise another set of 33 microarrays.

### RNA isolation

Total RNA was extracted from 64 samples using the RNeasy Mini kit (Qiagen). RNA quantity and integrity were determined by the 2, 100 Bioanalyzer (Agilent Technologies). Using the RIN algorithm (RNA Integrity Number) as a quality standard, we established a threshold of 5.6 for the selection/inclusion of samples in the study [21,22].

### RNA labelling and array hybridisation

RNA labelling and hybridisation were performed following the Agilent Low RNA Input Fluorescent Linear Amplification kit and Agilent In situ Hybridization kit-plus. Briefly, reverse transcription was performed on 500 ng of total RNA to synthesize the first and second strands of cDNA. Next, the cRNA was synthesized by T7 RNA polymerase which simultaneously incorporates the fluophores. The pool was labelled with Cyanine-3-CTP (PerkinElmer), whereas the tumoral and non-tumoral samples were labelled with Cyanine-5-CTP (PerkinElmer). Labelled probes were measured in the spectrophotometer at 550 nm for the samples labelled with Cy3 and at 650 nm for the ones labelled with Cy5. In order to determine the efficiency of the labelling reaction, the ratio of the picomoles of cyanine dye per μg of cRNA was calculated. Only those labelled samples with a ratio between 10 and 20 were hybridised.

0.75 μg of labelled cRNA from each sample were hybridised onto Human 1A Oligo Microarrays 22 K (Agilent Technologies). The arrays were placed inside the hybridisation oven (Agilent Technologies) and hybridisation reaction was performed for 17 h at 60°C and 4 rpm. The slides were then washed at room temperature in 20X SSPE and 20% N-laurylsarcosine and dried with the Stabilization and Drying Solution (Agilent Technologies).

### Scanning and image process

The hybridised arrays were scanned using the GenePix 4000B dual laser slide scanning system (Axon Instruments). The images were processed with the GenePix Pro 6.0 (Axon), with 10 μm resolution. Microarray internal controls named 'N/A', 'NegativeControl', 'BrightCorner', 'Pro25G' and 'eQC' were removed prior to normalisation. Microarray data are available in http://www.ebi.ac.uk/arrayexpress under the accession number E-MTAB-476.

### Probes quality pre-processing

#### Probe quality metrics

Following the quality metrics defined by Chen *et al. *[23] we computed three probe quality criteria: fluorescent intensity measurement, background flatness and signal intensity consistency qualities. Values of each metric vary between 0, the lowest, and 1, the maximum. In order to get the most reliable readings, the global quality metric for a given probe *k *was defined as the minimum value of the previous three. In our case, the acceptance threshold was set up in an average of 0.99 and a total of 11, 120 probes surpassed this stage.

#### Normalisation

All the readings coming from the probes that surpassed the quality criteria were smoothed by means of the Lowess technique [24]. Lowess normalisation assumes that the data bias is dependent on spot intensity. The *logRatio *values are then adjusted subtracting the lowess fit to the MA-plot from the original logRatio values.

#### Lost values imputation

Among all the available imputation methods for missing values, the one that is more broadly used in the microarray field is the *kNN-Impute *[25]. In this method, the classical machine learning *k nearest neighbours *algorithm is adapted to microarray data. The imputation algorithm for our data was run with a *k *value of 15 neighbours. From the total number of spots, there were only 1.04% of lost values (7, 534 probes) to imputate.

#### Intra-class variability

**Figure 1:**
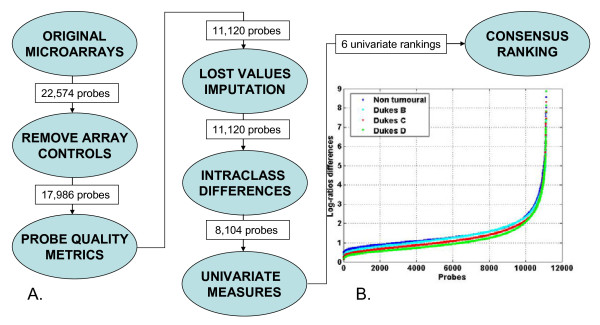
**A) Overall data analysis workflow**. B) Intraclass dispersion measure for the 11, 120 filtered quality probes.

A side effect of working with biopsies is that there are different types of tissues within each sample. This could lead to great differences in the expression profiles of particular genes. Thus, the last filtering step dealt with the assessment of intra-class variability. We removed 3, 016 probes that showed differences greater than 2-fold between each of the four classes of tissues (non-tumoral, Dukes B, C and D stages). Therefore, the final dataset was composed of a total of 8, 104 probes and 64 cases or microarrays. Figure 1 shows the workflow carried out during the data filtering process.

### Statistical analysis

#### Analysis of the univariate relevance of each gene

There are several approaches for identifying differentially expressed genes [26]. One common approach is to look for those genes that show a high differential expression between phenotypes. In order to identify such representative set of genes, we firstly performed an ensemble of univariate relevance rankings. Six non-parametric relevance metrics were measured: Mutual information, Euclidean distance, Matusita distance, Kullback-Leibler divergence (with two formulations) and Bhattacharyya metric. Each of them provided a relevance ranking of the 8, 104 genes and these rankings were combined into an ensemble one as a problem of aggregation of individual preferences [27] (Table 1).

**Table 1:** Consensus ranking of the 8,104 probes. In this table the first 50 genes are represented.

Ranking Position	Agilent ID	Gene	Description	Access Number
1	A_23_P213424	*ENC1*	Ectodermal neural cortex 1 (with BTB-like domain)	NM_003633

2	A_23_P24515	*ACAT1*	Acetyl -coenzyme A acetyltransferase 1	NM_000019

3	A_23_P24716	*TMEM132A*	Transmembrane protein A	NM_017870

4	A_23_P40309	*SNRPB2*	Small nuclear ribonucleoprotein polypeptide B	NM_003092

5	A_23_P13663	*FAM60A*	Family with sequence similarity 60, member A	NM_021238

6	A_23_P142872	*TCF7L1*	Transcription factor 7-like 1	NM_031283

7	A_23_P47843	*DDX55*	DEAD(Asp-Glu-Ala-Asp) box polypeptide 55	NM_020936

8	A_23_P114282	*MCTS1*	Malignant T cell amplified sequence 1	NM_014060

9	A_23_P103149	*ACO2*	Aconitase 2	NM_001098

10	A_23_P207999	*PMAIP1*	Phorbol-12-mystirate-13-acetate-induced protein 1	NM_021127

11	A_23_P63584	*AHCTF1*	AT hook containing transcription factor	NM_015446

12	A_23_P22086	*LOC649828*	Similar to adenosylhomocysteinase hidrolase (S-adenosyl-L-homocysteine)	NW_927818

13	A_23_P88522	*NMB*	Neuromedin B	NM_021077

14	A_23_P256413	*CMTM7*	CKLF-like MARVEL transmembrane domain containing 7	NM_138410

15	A_23_P153615	*MADCAM1*	Mucosal vascular addressin cell adhesion molecule 1	NM_130760

16	A_23_P112412	*TEX10*	Testis expressed 10	NM_017746

17	A_23_P209070	*LG14*	Leucine rich repeat LGI family	BC087848

18	A_23_P123343	*NUDCD1*	NudC domain containing 1	NM_032869

19	A_23_P163179	*CALM1*	Calmodulin 1 (phosphorilase kinase, delta)	NM_006888

20	A_23_P253412	*MRLP50*	Mitochondrial ribosomal protein L50	NM_019051

21	A_23_P34018	*RPL39*	Ribosomal protein L30	NM_001000

22	A_23_P70827	*KIAA1549*	Unknown function	AL136736

23	A_23_P113634	*CBFB*	Core-binding factor beta, subunit of dimeric polyomavirus enhancer binding transcription factor	NM_001755

24	A_23_P30464	*PRR7*	Proline rich 7 (Synaptic)	NM_030567

25	A_23_P37375	*RPS6KA5*	Ribosomal protein S6 kinase A5	NM_004755

26	A_23_P48771	*C14orf159*	Chromosome 14 open reading frame 159	NM_024952

27	A_23_127175	*SAR1A*	SAR gene homolog A	NM_020150

28	A_23_P141180	*TOM1L2*	Target of myb-like 2	AK055959

29	A_23_P69399	*MST1*	Macrophage stimulating 1 protein	NM_020998

30	A_23_131846	*SNAI1*	Snail 1 homolog	NM_005985

31	A_23_P121657	*HS3ST1*	Heparan sulphate D-glucodaminyl 3-O-sulfotransferase	NM_005114

32	A_23_P33027	*MLXIP*	MLX interacting protein	NM_014938

33	A_23_123343	*NUDCD1*	NudC domain containing 1	NM_032869

34	A_23_P108676	*TMEM166*	Transmembrane protein 166	NM_032181

35	A_23_103201	*PNRC2*	Proline rich nuclear transcription co-activator 2	NM_017761

36	A_23_P51269	*CD641036*	Protein with high similarity to BTF3	CD641036

37	A_23_P123330	*RPL30*	Ribosomal protein L30	NM_000989

38	A_23_P252118	*POLB*	DNA polymerase beta	NM_002690

39	A_23_P215517	*KLHL7*	Kelch-like 7	BC00955

40	A_23_P145194	*BYSL*	Bystin-like protein	NM_004053

41	A_23_P134274	*POP7*	Processing of precursor 7, ribonuclease protein subunit	NM_005837

42	A_23_P70915	*ORAI2*	ORAI calcium release-activated calcium modulator 2	NM_032831

43	A_23_P156890	*TCF21*	Transcription factor 21	NM_003206

44	A_23_P51906	*PFDN2*	Prefoldin subunit 2	NM_012394

45	A_23_P114232	*PRDX4*	Peroxiredoxin 4	NM_006406

46	A_23_P215525	*OSBPL3*	Oxysterol binding protein like 3	NM_015550

47	A_23_P27867	*PLAUR*	Plasminogen activator, urokinase receptor	NM_002659

48	A_23_P157405	*CHCHD2*	Coiled-coil-helix-coiled domain containing 2	NM_016139

49	A_23_P118102	*NDUFB10*	NADH dehydrogenase (ubiquinone) 1 beta subcomplex	NM_004548

50	A_23_P78423	*ATP5A1*	ATP synthase H + transporting mitochondrial F1	NM_001001937

In this table the first 50 genes are represented

#### Multivariate gene analysis by means of ensemble of classification models

**Figure 2:**
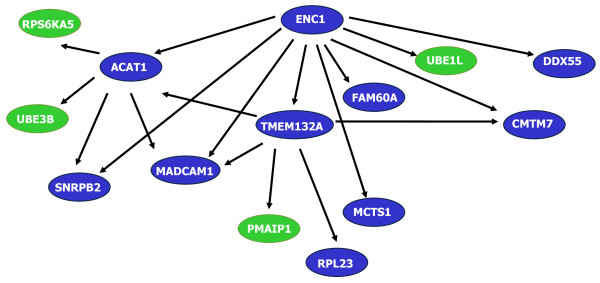
**Graphical structure of the high-reliable dependences network**. The occurrence threshold was set in 100 out of the total 1, 000 main bootstrap iterations. The constant presence of this core of genes gives the criterion to select them as the final biomarker panel.

Univariate approaches are to some extent limited and they ignore the role of gene combinations that could provide a good classification. Therefore, our next step consisted on the selection of a subgroup of relevant genes that were able to distinguish tumour from non-tumour samples. This process is known as feature subset selection (FSS) and it is still a growing discipline [28]. Recent studies show that as important as the classification accuracy is the behaviour of the FSS approach: stable approaches should be used instead of those with a great degree of variance [29]. To accomplish this task, we performed the ensemble approach proposed by Armañanzas *et al. *[30]. This data mining approach makes use of random resampling of the dataset, a multivariate feature subset selection and the induction of a *k*-dependence Bayesian network classifier [31]. To seek for the most stable output, the process is repeated a significant number of times in order to reduce the presence of false positives findings. The output of the method is comprised by an ensemble of all the induced networks, that is, a set of high reliability gene expression relationships. This methodological proposal includes a set of running parameters to be fixed. Especially in the microarray domain, all these parameters are expected to set a scenario in which the running time could be affordable. Correlation feature selection (CFS) [32] has been widely used in this bioinformatics context, reporting good results both in time and in relevant genes [27]. CFS addresses two fundamental issues, avoid redundancy and irrelevancy in the selected subset of features. Therefore CFS is configured as the internal selector of relevant genes. Gene expression data may provide information about various relationships between genes, which often can be viewed as networks. Hence, using the filtered data, high confiability Bayesian networks were carried out. We performed 1, 000 random samplings of our database and in each of them we applied the CFS technique, inducing a *k*-dependence Bayesian classifier (kDB) for each intermediate dataset (reduced to the genes found by CFS) each time [31]. This way, the genes most times configured throughout the structures were identified. Table 2 shows a list of the 20 most recurrent genes obtained. The most robust statistical dependences obtained from the whole experiment were jointly gathered into a Bayesian network (Figure 2).

**Table 2:** **Selected genes. **This table represents the first 20 probes identified as the most selected ones.

Times selected	Agilent ID	Gene	Description	Access Number
961	A_23_P24515	*ACAT1*	Acetyl -coenzyme A acetyltransferase	NM_000019

820	A_23_P213424	*ENC1*	Ectodermal neural cortex 1 (with BTB-like domain)	NM_003633

567	A_23_P24716	*TMEM132A*	Transmembrane protein A	NM_017870

547	A_23_P256413	*CMTM7*	CKLF-like MARVEL transmembrane domain containing 7	NM_138410

512	A_23_P7353	*LARP2*	La ribonucleotide domain family member 2	NM_178043

435	A_23_P153615	*MADCAM1*	Mucosal vascular addressin cell adhesion molecule 1	NM_130760

330	A_23_P26717	*RPL23*	Ribosomal protein L23	NM_000978

310	A_23_P13663	*FAM60A*	Family with sequence similarity 60, member A	NM_021238

287	A_23_P47843	*DDX55*	DEAD(Asp-Glu-Ala-Asp) box polypeptide 55	NM_020936

275	A_23_P13102	*CASP12*	Caspase 12	NM_014383

273	A_23_P35521	*P4HA1*	Proline 4-hidroxylase alpha plypeptide	NM_000917

267	A_23_P27964	*HOMER-3*	Homer neuronal immediate early gene 3	NM_004838

249	A_23_P40309	*SNRPB2*	Small nuclear ribonucleoprotein polypeptide B	NM_003092

246	A_23_38262	*ELAC2*	ElaC homolog 2	NM_018127

238	A_23_P113634	*CBFB*	Core-binding factor beta, subunit of dimeric polyomavirus enhancer binding transcription factor	NM_001755

233	A_23_P210253	*DGKD*	Diacylglycerol kinase delta	NM_003648

221	A_23_P33075	*LO8961*	Similar to c-Mer protooncogen tyrosine kinase (human MERTK)	LO8961

221	A_23_P131846	*SNAI1*	Snail 1 homolog	NM_005985

218	A_23_P206268	*TRAPPC2L*	Trafficking protein particle complex 2-like	NM_016209

208	A_23_P35125	*SF3B4*	Splicing factor 3b (SF3B) subunit 4	NM_005850

This table represents the first 20 probes identified as the most selected ones

From the visualization of the Bayesian network, we selected 10 genes with an altered expression between tumoral and non-tumoral samples.

### Quantitative real-time PCR

Quantitative real-time PCR is a commonly used validation tool for confirming gene expression results obtained from microarray analysis. Expression levels of the differentially expressed genes were measured using TaqMan reverse transcription reagents (Applied Biosystems) and Platinum Quantitative PCR Supermix-UDG with ROX (Invitrogen). The reaction was performed in MyiQ Single-Colour Real-Time PCR Detection System (Biorad) as follows: 1 cycle at 95°C for 2 min, following 40 cycles of 95°for 10 s and 60°for 1 min. Results were analysed with the MyIQ™ (Biorad) software to obtain the C_q _values for each sample. A ΔC_q _value was calculated reflecting the difference between the average C_q _of the replicate samples obtained for the control gene (18S) and the average C_q _of the replicate samples obtained for the test gene to be validated. Using these ΔC_q _values as the raw expression value in the qPCR experiment, we first determined the median ΔC_q _for all the non-tumoral control samples. Next, we calculated the difference between the ΔC_q _of each test sample and the ΔC_q _values of the controls, thus obtaining a set of ΔΔC_qdiff _values for each gene.

### Machine learning validation using a new cohort of samples

In order to validate the classification power of the biomarker panel, we tackled the classification of a new cohort of samples, but using only the expression of the genes previously identified within the panel [33]. The new microarrays were produced following the same protocols and technologies presented in previous sections. There were a total of 36 tissue samples: 14 non-tumoral, 2 stage A, 11 stage B, 3 stage C and 6 stage D (Additional file 2). To follow the rationale of the paper, all the 22 tumoral samples were grouped together into a single category labeled tumoral. All the 36 samples (14 non-tumoral and 22 tumoral) were hybridised against the same reference pool previously described (see *Experimental Design *section). These microarray data are available in http://www.ebi.ac.uk/arrayexpress under the accession number E-MTAB-770.

Three different classification paradigms were used to validate the panel: naïve Bayes (nB), support vector machines (SVM) and *k*-nearest neighbors (*k*-NN) with *k *= 3 to avoid ties [34]. In order to assess the classification performance of each of the mentioned paradigms, we used a leaving-one-out cross-validation scheme (LOOCV). This validation scheme estimates the accuracy of a given classification model by inducing the same number of classifiers as the dataset is comprised of. Each model is built with all the cases but one and tested on the left out case. The model accuracy is finally estimated as the average accuracy over all these classifiers. In addition to the estimated classification accuracy, the area under the receiving operating characteristic (ROC) curve or AUC was also considered.

### Biological pathway analysis

As an exploratory approach, we used Ingenuity Pathways Analysis (IPA) software (Ingenuity^® ^Systems, http://www.ingenuity.com) to assess the involvement of the relevant genes in known molecular pathways and networks. Briefly, a dataset containing the gene names was uploaded into the software. Networks were generated as graphical representations of the molecular relationships between genes. Genes are represented as nodes and the biological relationship between two nodes is represented as an edge (line). Nodes are displayed with various shapes that represent the functional class of the gene product. Right-tailed Fisher's exact test was used to calculate a *p*-value determining the probability that each biological function and/or disease assigned to our dataset was due to chance alone.

## Results

The graphical dependency structure reported in Figure 2 gathers a total of 14 genes with an occurrence threshold greater or equal than *t *= 100. From all of them, ***ENC1 ***(ectodermal-neural cortex, with BTB-like domain) results in a core gene that shows dependences with 9 out of the 14 genes represented. This gene, also known as *NRPB *or *PIG10*, is a peptidase that regulates the expression of *CEACAM5, CASP3 *or *Erk1/2 *[35]. It also binds to actin and retinoblastoma 1 (*RB1*) gene [36]. Regarding its role in colon cancer, Fujita *et al. *[37] suggested that *ENC1 *is regulated by β-catenin/TCF pathway and its altered expression may contribute to colorectal carcinogenesis by suppressing differentiation of colonic cells. In the downstream dependences, we find ***ACAT1 ***(acetyl-coenzyme A acetyltransferase 1), which has been shown to play a pivotal functional role in the intestinal absorption of cholesterol, hepatic secretion of VLDL, biosynthesis of steroid hormones, production of cholesterol esters in macrophages in atheroma and secretion of biliary cholesterol [38]. Next, ***TMEM132A ***(transmembrane protein 132A) has been described as an important factor of cell survival in regulating certain endoplasmic reticulum stress-related gene expression in neuronal cells [39]. It has not been related to carcinogenesis or cancer progression as yet. ***MADCAM1 ***(mucosal vascular addressing cell adhesion molecule 1) is preferentially expressed in endothelial cells of the intestinal mucosa, submucosa and Peyer's patches [40]. High expression of this and other adhesion molecules has been correlated with prolonged disease-free survival in CRC [41]. It interacts preferentially with the leukocyte beta7 integrin LPAM-1 (alpha4beta7), L-selectin, and VLA-4 (alpha4beta1) on myeloid cells to direct leukocytes into mucosal and inflamed tissues. Two other reported genes, ***MCTS1 ***(malignant T cell amplified sequence 1) and ***SNRPB2 ***(small nuclear ribonucleoprotein polypeptide B) have been related to proliferation. *MCTS1 *was considered as a possible oncogene [42], and *SNRPB2 *is an essential component of the mRNA splicing machinery playing a role in cell proliferation [43,44].

Although not directly influenced by *ENC1*, we identified ***RPL23 ***(ribosomal protein L23). This gene regulates *TP53 *[45] among other genes and it takes part in G1/S phase transition.

Finally, the Bayesian model also revealed other recurrent genes, ***FAM60A ***(family with sequence similarity 60, member A), ***CMTM7 ***(CKLF-like MARVEL transmembrane domain 7) and ***DDX55 ***(DEAD (Asp-Glu-Ala-Asp) box polypeptide), currently not reported to be involved in cancer progression.

**Figure 3:**
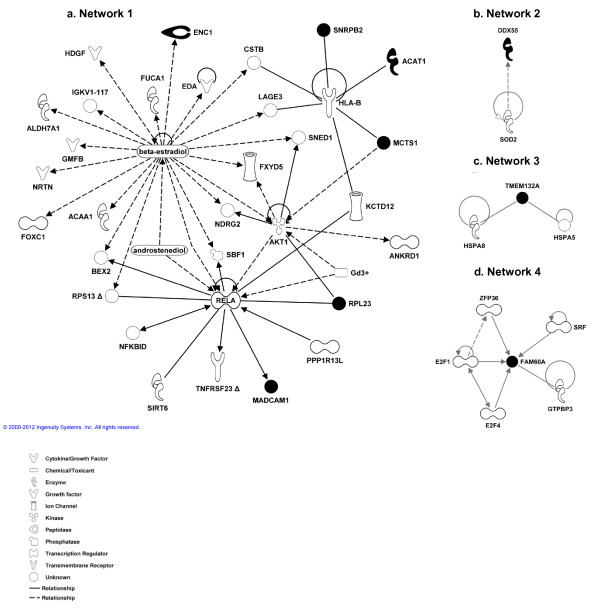
**Main networks obtained for the most relevant genes**. These networks were generated through the use of Ingenuity Pathways Analysis www.ingenuity.com. Genes are represented as nodes and the biological relationships between 2 nodes is represented as an edge (line). The functional analysis of the networks identified the biological functions and/or diseases that were most significant to the molecules in each network. In this sense, network 1 is associated to Cancer, Genetic disorder and Reproductive system disease; network 2 is associated to Free radical scavenging, Metabolic disease and Renal and Urological Disease; network 3 is associated to Post-translational Modifications, Protein folding and Cell death and network 4 is associated to Skeletal and Muscular System Development and Function, Tissue Development and Cell Death. Molecules in black represent the genes from our dataset. Dash lines represent indirect relationships (2000-2010 Ingenuity Systems, Inc).

These 10 genes were subjected to IPA software, where they were mapped to networks defined by Ingenuity's database (Figure 3). Therefore, 6 of the genes (*ENC1, ACAT1, MSTC1, MADCAM1, RPL23 *and *SNRPB2*) were found to be associated with cancer, genetic disorder or reproductive system disease within the same network. The rest of the genes were involved in different networks, with dissimilar biological functions associated to them: the *DDX55 *gene is related to free radical scavenging, metabolic disease and renal and urological disease; *TMEM132A *is linked to post-translational modification, protein folding and cell death and *FAM60A *is connected to skeletal and muscular system development and function, tissue development and cell death. *CMTM7 *involvement in any biological process has not been reported yet.

### Experimental validation by qRT-PCR

This technique provides quantitative assessment of the relative abundance of specific transcripts using gene-specific primers [46]. To examine the reliability of our microarray results, we did a qPCR experiment with the genes identified. This validation was carried out with 15 new samples (4 non-tumoral and 11 tumoral samples). For each gene and phenotype, ΔΔC_qdiff _values were calculated (see *Methods*), and the median values of these ΔΔC_qdiff_, together with the expected gene expression activities are shown in Table 3. As a dispersion measure of the results, the values for the first and third quartile of each group of values are also shown. These results confirmed that the expected gene expression profiling as measured by microarray quantitation is validated by the qPCR experiment for 7 of the genes analysed.

**Table 3:** qPCR output values and expected activity for 7 genes from the relevant gene list

Gene Symbol	Median	1st-quartil	3rd-quartil	Expected
**ENC1**	2.12	1.71	3.19	UP

**ACAT1**	-1.63	-1.99	-0.83	DOWN

**TMEM132A**	2.80	2.42	3.63	UP

**CMTM7**	1.65	0.19	2.58	UP

**FAM60A**	0.93	0.34	1.35	UP

**MADCAM1**	-2.48	-5.10	-0.21	DOWN

**DDX55**	1.11	0.53	1.28	UP

### Machine learning validation by different classification paradigms

The pre-processing of the new cohort of 36 microarrays was done in accordance with the protocols also used in the original arrays. After reducing the data matrix to just the 10 genes gathered in the panel of biomarkers, a LOOCV using three different classification paradigms was performed. The performance of the gene set was outstanding with an estimated accuracy of 94.45% and AUC of 0.994, 0.995 and 0.971 for nB, SVM and *k*-NN, respectively. In order to test the subset of genes that had already passed the qPCR validation stage, we retained only those seven genes (see Table 3) and redid the experiment. The results in this case reported the same estimated accuracy of 94.45% with very slight differences in terms of AUC (0.997, 0.955 and 0.974 for nB, SVM and *k*-NN respectively). Specially relevant is the fact that for every of these estimations the specificity score was 1, i.e. the false positive rate (FPR) was zero. In terms of the confusion matrix, this fact implies that all the non-tumoral samples were always correctly classified as non-tumoral, whereas just two of the tumoral samples were classified as non-tumoral (sensitivity score of 0.909).

## Discussion

Sporadic CRC is one of the most frequent types of cancer in our society and current treatment has the best effect on the early-stage disease. Colorectal carcinogenesis involves a network of genetic alterations that affect DNA repair genes, oncogenes and tumour suppressor genes. An important objective in nowadays research is the discovery of new biomarkers that can detect colon tumours in early stages. Recently, molecular studies have extended the opportunity for testing new potential markers as only a few markers can be recommended for practical use in clinic [47]. Gene expression analysis studies have resulted in many new insights in cancer biology and mRNA expression analysis is turning out to be a very useful tool for cancer classification, cancer diagnosis and disease outcome prediction [48].

The main objective of our study was to develop a tentative model for the classification of non-tumoral and tumoral samples, based on their expression profiles. One important requirement in the screening of CRC is that the test used should have high sensitivity and specificity, namely, a low number of false-negative and false-positive results. Our approach combined a resampling method with an inner feature selection technique and a Bayesian *k-dependence *classifier to obtain a relevant gene subset. By means of this gene panel, different supervised classifiers were induced to classify 36 newly unseen samples. To estimate the performance of the models, a *leave-one-out *cross validation (LOOCV) [49] was performed. The estimation achieved a 94.45% degree of accuracy with associated AUC values between 0.997 and 0.955, with only two misclassified samples in the confusion matrix (specificity = 1, sensitivity = 0.909 using only the 7 genes validated by the qPCR). Non-tumoral samples were always distinguished from the tumoral ones. These results over a set of unseen samples flawlessly support the joint classification ability of the identified biomarker panel found using the initial cohort of samples

**Figure 4:**
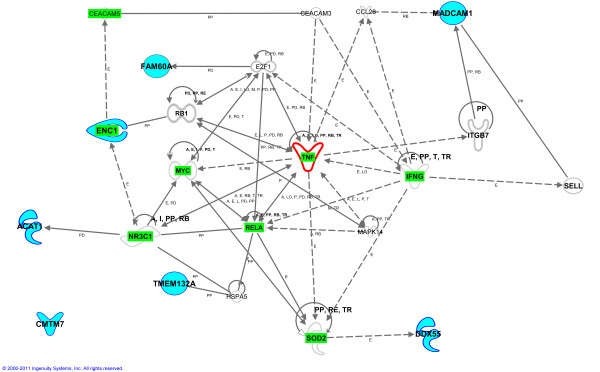
**Molecular relationships between genes**. This network is a graphical representation of the relationships between the validated genes obtained from the analysis and other molecules. Molecules in blue represent the 7 validated genes (see legend in figure 4) Genes involved in colon cancer are highlighted in green. Only human relationships are presented. All edges are supported by at least 1 reference from the literature, textbook or canonical information stored in Ingenuity's Knowledge Base (2000-2011 Ingenuity Systems, Inc).

We used Ingenuity Pathways Analysis software (Ingenuity^® ^Systems, http://www.ingenuity.com) in an attempt to decipher the involvement of these genes in biological networks. Although no direct relationships were found between our set of validated genes, all of them, except for *CMTM7*, were involved in a network were *TNF *(tumour necrosis factor) and secondly *RELA *(v-rel reticuloendotheliosis viral oncogene homolog A (avian)) played a central part (Figure 4). Both genes are related to inflammatory processes and take part in CRC metastasis canonical pathway [50]. According to IPA's Knowledge Base functional analysis, from the 19 genes implicated in this network, 14 are involved in cancer with a p-value of 1.14E-05. Among them, *ENC1 *and *ACAT1 *are directly involved in this disease as previously mentioned [33,37,51]. Expression of *ENC1 *was examined in colon cancer samples and their corresponding non-cancerous tissues using semiquantitative RT-PCR, and its expression was increased in 17 of the 24 tumours analysed [37]. These results subscribe our findings in microarray analysis, where this gene showed an upregulation in tumoral samples, comparing to the non-tumoral counterparts. Moreover, Figure 4 shows that *ENC1 *interacts with *RB1 *(retinoblastoma 1) [36], which acts as a tumour suppressor gene through the regulation of transcription of *MYC *(v-myc myelocytomatosis viral oncogene homolog (avian)) and other genes involved in growth [52]. Regarding *ACAT1*, it has been proposed that it is constitutively expressed and likely functions to maintain the intracellular balance of free and esterified cholesterol [53]. In our microarray analysis we observed that this gene was downregulated in tumoral samples. Ancona *et al. *performed a microarray analysis and observed that *ACAT1 *was downregulated in tumoral tissue, comparing to normal mucosa [33].

Others, as *TMEM132A *or *MADCAM1 *are indirectly involved in tumorigenesis through protein-protein interactions with *HSPA5 *(heat shock 70 kDa protein 5 (glucose-regulated protein, 78 kDa)) and *SELL *(selectin L) respectively. *FAM60A *is also implicated in the tumorigenesis process through protein-DNA interaction with *E2F1 *(E2F transcription factor 1) and finally, *DDX55 *expression is indirectly controlled by *SOD2*(superoxide dismutase 2, mitochondrial), which is directly involved in neoplasia and carcinogenesis. Furthermore, from the 14 genes that IPA found to be involved in cancer, 8 genes are specifically related to CRC, with a *p*-value of 7.22E-06 (Table 4) and our Bayesian model's core gene, *ENC1*, among them.

**Table 4:** Genes involved in cancer and colorectal cancer diseases

Disease	*p*-value (Fisher's exact test)	Genes involved
Cancer	1.14E-05	*CEACAM5, E2F2, RB1, ENC1, ACAT1, NR3C1, MYC, HSPA5, SOD2, RELA, MAPK14, IFNG, TNF, SELL*

Colorectal cancer	7.22E-06	*CEACAM5, ENC1, MYC, TNF, IFNG, RELA, NR3C1, SOD2*

Finally, Figure 4 shows that there are direct or indirect molecular relationships between the other genes from the panel and those involved in cancer. However, until now no public data has been reported regarding the role of these genes in cancer progression. Nevertheless, the whole gene panel (7 genes) is required as a group to identify new unseen samples as tumoral or non-tumoral with 96.92% accuracy.

## Conclusions

Consensus approaches are alternative techniques that try to overcome the technology intrinsic data noise in microarray experiments. In the present paper, we applied a supervised consensus gene selection method, aiming to add robustness to the biomarker identification procedures by means of DNA microarrays. Throughout this paper, from the starting feature selection to the final biological validations, we have exposed a battery of techniques, to add reliability and proofs to the results. We would like to emphasize the posterior validation of the findings by means of qPCR analysis with an extra set of samples not used in the previous statistical stage.

In conclusion, we have achieved a tentative biomarker consisting in a panel of 7 genes capable of correctly classifying cancerous and non-cancerous colon samples. This biomarker could constitute the basis of a new tool with high potential for CRC diagnosis. Future work will comprise the application of this pipeline to the analysis of each severity stage independently.

## Competing interests

As a result of this study, a patent has been filed on the use of this panel as a diagnostic tool in colorectal cancer (EP2169078A1). AGB, RA, II, PL, GLV, BSM and MB are inventors; ZI, AAV and BC declare that they have no competing interests.

This study was not supported by any company of commercial fund.

## Authors' contributions

AGB carried out the microarray studies, performed the biological interpretation of the data and drafted the manuscript. RA participated in the design of the experiment, carried out the data analysis and helped to write the manuscript. ZI participated in the microarray and validation studies, BC participated in validation studies, AAV contributed in writing the manuscript, II and PL participated in the design of the experiment and data analysis, GLV provided tumour material and clinical data and helped to write the manuscript, BSM contributed in the biological interpretation and drafted the manuscript. MB conceived the study, and participated in its design and coordination, biological interpretation and drafted the manuscript. All authors revised the manuscript and approved the final version.

## Pre-publication history

The pre-publication history for this paper can be accessed here:

http://www.biomedcentral.com/1471-2407/12/43/prepub

## Supplementary Material

Additional file 1 **Clinical sampled data**. M = male; F = female. Click here for file

Additional file 2 **New cohort of samples (clinical data)**. Click here for file
