# Supplementary material for: Identification of a biomarker panel for colorectal cancer diagnosis
Source: BMC Cancer. 2012 Jan 26;12:43. doi: 10.1186/1471-2407-12-43 (PMC3323359; doi:10.1186/1471-2407-12-43)
Supplement: Additional file 1 — Clinical sampled data. M = male; F = female. [file 1471-2407-12-43-S1.DOCX]

| PATIENT | TNM STAGE | DUKES’ STAGE (Astler & Coller) | AGE | SEX | RIN |
| --- | --- | --- | --- | --- | --- |
| 1 | IV | D | 46 | F | 8.6 |
| 2 | IIA | B2 | 69 | M | 7.5 |
| 3 | IIB | B3 | 68 | M | 5.8 |
| 4 | IV | D | 63 | M | 6.8 |
| 5 | IIIA | C1 | 87 | F | 7.6 |
| 6 | IIIC | C2 | 68 | F | 8.7 |
| 7 | IV | D | 81 | M | 9.1 |
| 8 | IV | D | 77 | F | 8.9 |
| 9 | IA | B1 | 73 | M | 8.3 |
| 10 | IIIC | C3 | 47 | M | 6.7 |
| 11 | IIA | B2 | 71 | F | 7.8 |
| 12 | IIIB | C2 | 73 | F | 6.4 |
| 13 | IIA | B2 | 55 | M | 5.6 |
| 14 | IIA | B2 | 66 | F | 6.9 |
| 15 | IA | B1 | 77 | M | 7.2 |
| 16 | IA | B1 | 46 | M | 6 |
| 17 | IV | D | 50 | M | 6.5 |
| 18 | IIIB | C3 | 67 | F | 6.6 |
| 19 | IV | D | 83 | M | 7.3 |
| 20 | IV | D | 74 | M | 6.9 |
| 21 | IIIB | C2 | 71 | F | 8.4 |
| 22 | IIIB | C2 | 65 | M | 6.2 |
| 23 | IIA | B2 | 57 | M | 6.5 |
| 24 | IIA | B2 | 71 | M | 5.8 |
| 25 | IIA | B2 | 76 | F | 6.5 |
| 26 | IIIB | C3 | 57 | F | 8.3 |
| 27 | IIA | B2 | 63 | M | 6.7 |
| 28 | IIA | B2 | 75 | F | 7.3 |
| 29 | IV | D | 69 | F | 9.2 |
| 30 | IIIA | C1 | 62 | F | 6.5 |
| 31 | IIIB | C2 | 67 | M | 8.5 |

Additional file 1
