# Supplementary material for: Identification of a biomarker panel for colorectal cancer diagnosis
Source: BMC Cancer. 2012 Jan 26;12:43. doi: 10.1186/1471-2407-12-43 (PMC3323359; doi:10.1186/1471-2407-12-43)
Supplement: Additional file 2 — New cohort of samples (clinical data). [file 1471-2407-12-43-S2.DOCX]

Additional file 2

| PATIENT | TNM STAGE | DUKES’ STAGE (Astler & Coller) | RIN |
| --- | --- | --- | --- |
| 1 | IIIC | C | 8 |
| 2 | IIA | B2 | 6.3 |
| 3 | IIA | B2 | 6.8 |
| 4 | I | A | 5.8 |
| 5 | IV | D | 6.6 |
| 6 | IIA | B2 | 9.1 |
| 7 | IIA | B2 | 6.9 |
| 8 | IV | D | 6.9 |
| 9 | IIA | B2 | 7.5 |
| 10 | IIA | B2 | 6.4 |
| 11 | IV | D | 6.6 |
| 12 | IIIC | C | 7.1 |
| 13 | IIA | B2 | 6.1 |
| 14 | IIA | B2 | 8. |
| 15 | IIA | B2 | 7.7 |
| 16 | I | A | 7.8 |
| 17 | IV | D | 7.6 |
| 18 | IV | D | 7.4 |
| 19 | IIA | B2 | 8.5 |
| 20 | IIA | B2 | 8.5 |
| 21 | IIIC | C | 7.4 |
| 22 | IIIB | D | 7.6 |
